# Fatigue Monitoring Through Wearables: A State-of-the-Art Review

**DOI:** 10.3389/fphys.2021.790292

**Published:** 2021-12-15

**Authors:** Neusa R. Adão Martins, Simon Annaheim, Christina M. Spengler, René M. Rossi

**Affiliations:** ^1^Empa, Swiss Federal Laboratories for Materials Science and Technology, Laboratory for Biomimetic Membranes and Textiles, St. Gallen, Switzerland; ^2^Exercise Physiology Lab, Institute of Human Movement Sciences and Sport, ETH Zurich, Zurich, Switzerland; ^3^Zurich Center for Integrative Human Physiology (ZIHP), University of Zurich, Zurich, Switzerland

**Keywords:** fatigue monitoring, wearable, occupational health and safety, signal quality assessment, validation, physiological signal, machine learning, imbalanced datasets

## Abstract

The objective measurement of fatigue is of critical relevance in areas such as occupational health and safety as fatigue impairs cognitive and motor performance, thus reducing productivity and increasing the risk of injury. Wearable systems represent highly promising solutions for fatigue monitoring as they enable continuous, long-term monitoring of biomedical signals in unattended settings, with the required comfort and non-intrusiveness. This is a p rerequisite for the development of accurate models for fatigue monitoring in real-time. However, monitoring fatigue through wearable devices imposes unique challenges. To provide an overview of the current state-of-the-art in monitoring variables associated with fatigue via wearables and to detect potential gaps and pitfalls in current knowledge, a systematic review was performed. The Scopus and PubMed databases were searched for articles published in English since 2015, having the terms “fatigue,” “drowsiness,” “vigilance,” or “alertness” in the title, and proposing wearable device-based systems for non-invasive fatigue quantification. Of the 612 retrieved articles, 60 satisfied the inclusion criteria. Included studies were mainly of short duration and conducted in laboratory settings. In general, researchers developed fatigue models based on motion (MOT), electroencephalogram (EEG), photoplethysmogram (PPG), electrocardiogram (ECG), galvanic skin response (GSR), electromyogram (EMG), skin temperature (T_sk_), eye movement (EYE), and respiratory (RES) data acquired by wearable devices available in the market. Supervised machine learning models, and more specifically, binary classification models, are predominant among the proposed fatigue quantification approaches. These models were considered to perform very well in detecting fatigue, however, little effort was made to ensure the use of high-quality data during model development. Together, the findings of this review reveal that methodological limitations have hindered the generalizability and real-world applicability of most of the proposed fatigue models. Considerably more work is needed to fully explore the potential of wearables for fatigue quantification as well as to better understand the relationship between fatigue and changes in physiological variables.

## Introduction

Fatigue, often defined as a decrement in mental and/or physical performance caused by cognitive overload, physical exertion, sleep deprivation, circadian phase/circadian rhythm disruption, or illness (International Civil Aviation Organization, 2012; Tanaka et al., 2015; Mohanavelu et al., 2017), has been a topic of research since the 1860ies. Various definitions of fatigue have been proposed in different scientific disciplines, however, none of them is applicable to all of these disciplines (Yung, 2016). Mainly the multidimensionality, interaction of different variables and in many cases, the subjective nature of perception of fatigue have not allowed to provide a unified definition.

Fatigue can develop in response to physiological challenges or pathophysiological changes. Fatigue has been classified according to its genesis as central (i.e., caused by impaired function of the central nervous system) or peripheral (i.e., caused by impaired function of the peripheral nervous or neuro-muscular system); but also according to the type of load as physical (i.e., of physical etiology, resulting from physical effort and leading to a decrease in physical performance) or mental (i.e., of psychological etiology, resulting from sustained cognitive activity and leading to a reduction in cognitive and behavioral performance) (Aaronson et al., 1999; Cavuoto and Megahed, 2016; Aryal et al., 2017). The latter classification is most commonly used, although other categorization methods have been proposed (Aaronson et al., 1999; Desmond and Hancock, 2001).

Fatigue quantification is of crucial relevance in areas such as occupational health and safety. Apart from being a physiological response of the human body possibly preventing it's overload, fatigue is a symptom associated with several diseases and health conditions (Casillas et al., 2006; Harris et al., 2009; Rudroff et al., 2016; Nassif and Pereira, 2018; O'Higgins et al., 2018; Cortes Rivera et al., 2019). Fatigue impairs cognitive and/or motor performance, reducing work efficiency, productivity, and product quality, as well as increasing risks for injury and fatality (Folkard, 2003; Cavuoto and Megahed, 2016; Yung, 2016). This fact renders fatigue a subject of utmost importance for work safety in occupational settings as, for instance, transportation, mining, aviation, or construction. As reported by police records, globally, 1–4% of the registered road crashes occur due to sleepiness and fatigue (Li et al., 2018). These estimates are nonetheless deemed to underrate the impact of fatigue on road safety, partially due to the inability to assess drivers' fatigue at the crash scene. Questionnaires, naturalistic observation studies, and in-depth investigation indicate that the actual value is around 10–20% of road crashes (European Commission, 2018). That share rises to 20–50%, when considering only commercial vehicle accidents (Davidović et al., 2018). Long working hours, and consequent fatigue and stress, were found to increase the hazard rate among US workers (Dembe, 2005). Furthermore, fatigue is involved in 4–8% of aviation mishaps (Caldwell, 2005).

These facts show the important role of continuous monitoring of the level of fatigue in an accurate and unobtrusive manner for early detection and management of fatigue. Among existing technologies, wearable sensors may meet these requirements. Wearables enable continuous, long-term monitoring, paving the way for the development of accurate models for fatigue monitoring in real-time. Therefore, their application for fatigue monitoring is highly promising.

Although several approaches have already been proposed for fatigue detection and monitoring, no gold standard measure of fatigue exists. Existing non-invasive methods are mainly based on five measuring principles: subjective measures, performance-related methods, biomathematical models, behavioral-based methods, and physiological signal-based methods of which not all are useful for online-monitoring during work or leisure.

### Subjective Measures

Subjective measures consist of assessing self-reported fatigue through questionnaires and scales (Shahid et al., 2010; Gawron, 2016). These are not useful in the context of online monitoring, however, they provide insights into mental and emotional processes underlying performance in a task. Subjective measures are therefore helpful as gold-standards to compare with results of fatigue models.

### Performance-Related Methods

Performance-related methods rely on the fact that subjects' cognitive and consequently motor performance on specific tasks reflects their level of fatigue. These methods consist of conducting tests to assess subjects' task performance, with emphasis on cognitive skills (e.g., vigilance, hand-eye coordination, sustained attention, reaction time) using neuro-behavioral tasks (Dawson et al., 2014; Honn et al., 2015). Despite being easy to standardize, also performance-related methods cannot be used to detect development of fatigue in real-time to allow preventive measures before an incident happens (Balkin et al., 2011; Huang et al., 2018; Zhang Y. et al., 2018).

### Biomathematical Models

Biomathematical models predict subjects' level of fatigue based on information regarding sleep-wake times, work-rest pattern as well as circadian cycle. They are typically used for fatigue risk assessment in civil and military aviation. These models have been developed based on data collected during partial and total sleep deprivation studies. To avoid reliance on self-reports and to increase the reliability of estimates of sleep/wake times, wrist-mounted actigraphy has been employed (Hursh et al., 2004).

Although biomathematical models predict fatigue with moderate success (Chandler et al., 2013), they have several limitations (Dawson et al., 2011) being based on cohort data, i.e., they are not able to predict fatigue at the individual level since individual needs for sleep, circadian rhythms, and responses to fatigue are different. Also, the type of task performed is not considered. To overcome this limitation, the inclusion of information regarding an individual, such as biological and psychological factors, and the performed task/context has been proposed (Chandler et al., 2013). Also, more recent models predict several additional performance- and sleep-related metrics but reliability of such predictions has not yet been proved (Bendak and Rashid, 2020).

### Behavioral-Based Methods

Behavioral-based methods follow an observational approach to detect fatigue and include external signs, such as yawning, sighing, eyes closure, or head nodding. As a result, technologies in this category frequently use metrics related to eye movements (EYE), head motion, and facial expression as input features (Dababneh et al., 2016; Sampei et al., 2016; Hu and Lodewijks, 2020). For this, computer vision technologies, especially cameras and eye trackers, have been widely employed for fatigue monitoring through this measuring principle. These solutions have been used in the transport and mining industries.

Although behavioral-based methods are not intrusive, allowing real-time fatigue monitoring almost without requiring faction from subjects, they detect fatigue only upon the appearance of its first signs, which may be too late to avoid exposure to fatigue-related risk (Balkin et al., 2011). Furthermore, computer vision technologies are sensitive to environmental factors (e.g., light).

### Physiological Signal-Based Methods

Physiological signal-based methods detect the onset of fatigue based on changes in subjects' physiological responses such as brain activity measured by electroencephalogram (EEG), heart rate (HR), or electromyogram (EMG). Electroencephalogram, for example, has been considered the gold standard for vigilance (Zhang et al., 2017; Zhou et al., 2018) and driver drowsiness detection (Hu et al., 2013; Sikander and Anwar, 2019; Hu and Lodewijks, 2020).

Taking physiological signals as indicators of fatigue enables objective, real-time fatigue monitoring at the individual level. However, changes in physiological variables in response to stressors and fatigue vary within and between individuals, which complicates the detection of abnormal conditions. Despite subjects having little control over their physiological signals, these are susceptible to several other factors, such as environmental conditions, emotions, and pathophysiological issues. For these reasons, the sensitivity and reliability of fatigue detection/prediction based on physiological signals, especially in real life settings, is still unclear (Balkin et al., 2011).

This work presents a review on the state-of-the-art in fatigue monitoring through wearable devices and associated measures of fatigue in addition to existing reviews on fatigue research that focus on specific application domains (e.g., Sikander and Anwar, 2019; Bendak and Rashid, 2020; Hu and Lodewijks, 2020) disregarding the type of technology used for fatigue quantification. Therefore, it is our purpose to provide an overview of fatigue monitoring approaches regardless of their application domain and the casual mechanisms behind fatigue, giving particular emphasis to the use of wearable technology. Strengths and weaknesses of current approaches to measure fatigue based on data acquired by wearable devices as well as limitations and existing opportunities in this field of research will be discussed.

## Methods

A literature search was performed in Scopus and PubMed databases covering the period from 1 January 2015 to 7 October 2020. Only articles in English, having the terms “fatigue,” “drowsiness,” “vigilance,” or “alertness” in their titles and the term “wearable” in any field were considered.

Inclusion criteria for this review were (a) studies proposing non-invasive, inter-subject approaches for fatigue quantification, (b) describing the methodology to develop the fatigue index or model, and (c) assessing model/index performance. Given the scope of this review, included studies should not merely propose wearable systems for fatigue monitoring, but also provide evidence to support their use for such application. Accordingly, studies investigating methods for fatigue quantification that did not use wearable systems to acquire data and those that explore the suitability of a given parameter or variable as a fatigue indicator without proposing a fatigue model/index or assessing its performance were dismissed.

A wearable device was defined as a small electronic device consisting of one or more sensors and a data logger intended to be worn on or attached to a single body location when the connection between sensors and logger is not wireless. The focus was on inter-subject, i.e., subject-independent, approaches due to their superior generalization ability compared to intra-individual approaches (i.e., based on data obtained from individual study participants).

No review protocol has been established. One reviewer inspected titles and abstracts of retrieved articles to identify potentially relevant publications. The full-text of the identified articles were then assessed for eligibility by two reviewers. Disagreements between reviewers were resolved by consensus. The following data were extracted from the selected articles: type of fatigue investigated and field of application; fatigue-inducing task; proposed approach to quantify fatigue and its performance metrics; reference measures of fatigue; wearable device used for data acquisition, variables monitored, and sensors' position; study design (i.e., in laboratory and/or real-world settings); and number of participants.

To assess the risk of bias of included studies, we have developed a component approach as recommended by the PRISMA statement (Liberati et al., 2009). This approach was founded on methodological aspects influencing models' or indices' performance. The contemplated aspects were identified based on existing empirical evidence and careful reasoning. Study characteristics used to support reviewers' judgment were selected by discussion and consensus among all review authors. A detailed description of the developed risk of the basis assessment tool as well as the rationale behind this is provided in Supplementary Table 1. In summary, we have considered a total of seven different components to assess the risk of bias of included articles:

1. Presence of a task to induce fatigue, named fatigue inducement.
2. Presence of validated fatigue reference measures, named validity of reference.
3. Application of strategies to handle imbalanced data distribution, if existing, named balanced dataset.
4. Application of strategies for cross-validation.
5. Validity of the wearable devices used to acquire the data, named validity of outcomes data. In the scope of this review, a device was considered valid if at least one validation study has been published in a peer-review journal, not exclusively authored by the device's developer or vendor (Byrom et al., 2018). A list of identified validation studies is provided in Supplementary Table 2.
6. Application of strategies to identify as well as to remove noise and artifacts from the acquired data, named signal quality assessment.
7. Number of participants included in the study. We have set the minimum acceptable number of participants in a study to 8 subjects.

The risk of bias judgments were made by one reviewer and checked by another. Studies were classified into three categories, low, unclear and high risk of bias, based on the components previously listed. Similarly to the Cochrane Collaboration's tool (Higgins et al., 2011), studies with low risk of bias in all components were deemed to be of low risk of bias, studies with low or unclear risk of bias for all components were deemed to be of unclear risk and those with a high risk of bias for one or more components were deemed to be of high risk of bias.

For the sake of clarity, selected articles were categorized according to the type of fatigue they address. Articles were grouped into four categories: mental fatigue, drowsiness, physical fatigue, or muscle fatigue (Williamson et al., 2011). The following definitions were applied:

1. Mental fatigue—decrease in mental performance as a result of cognitive overload (due to task duration and/or workload), independent of sleepiness (Borragán et al., 2017; Hu and Lodewijks, 2020).
2. Drowsiness—fatigue arising from sleep- and circadian rhythm-related factors (e.g., sleep deprivation, circadian rhythm disruption), monotony or low task workload (Hu and Lodewijks, 2020).
3. Physical fatigue—decline in overall physical performance caused by physical exertion (Zhang et al., 2019a).
4. Muscle fatigue—decrease in an isolated muscle performance due to reduced contractile activity (Allen et al., 2008; Wan et al., 2017).

Hereafter, the term *fatigue* presented in italic refers to fatigue in general. Articles addressing vigilance detection were considered as well but did not define an own category. This is because vigilance has been defined as the ability to sustain attention over prolonged time periods (Oken et al., 2006), and decrements in vigilance are strongly associated with *fatigue* onset but it cannot be considered as an individual type of *fatigue*.

## Results

**Figure 1:**
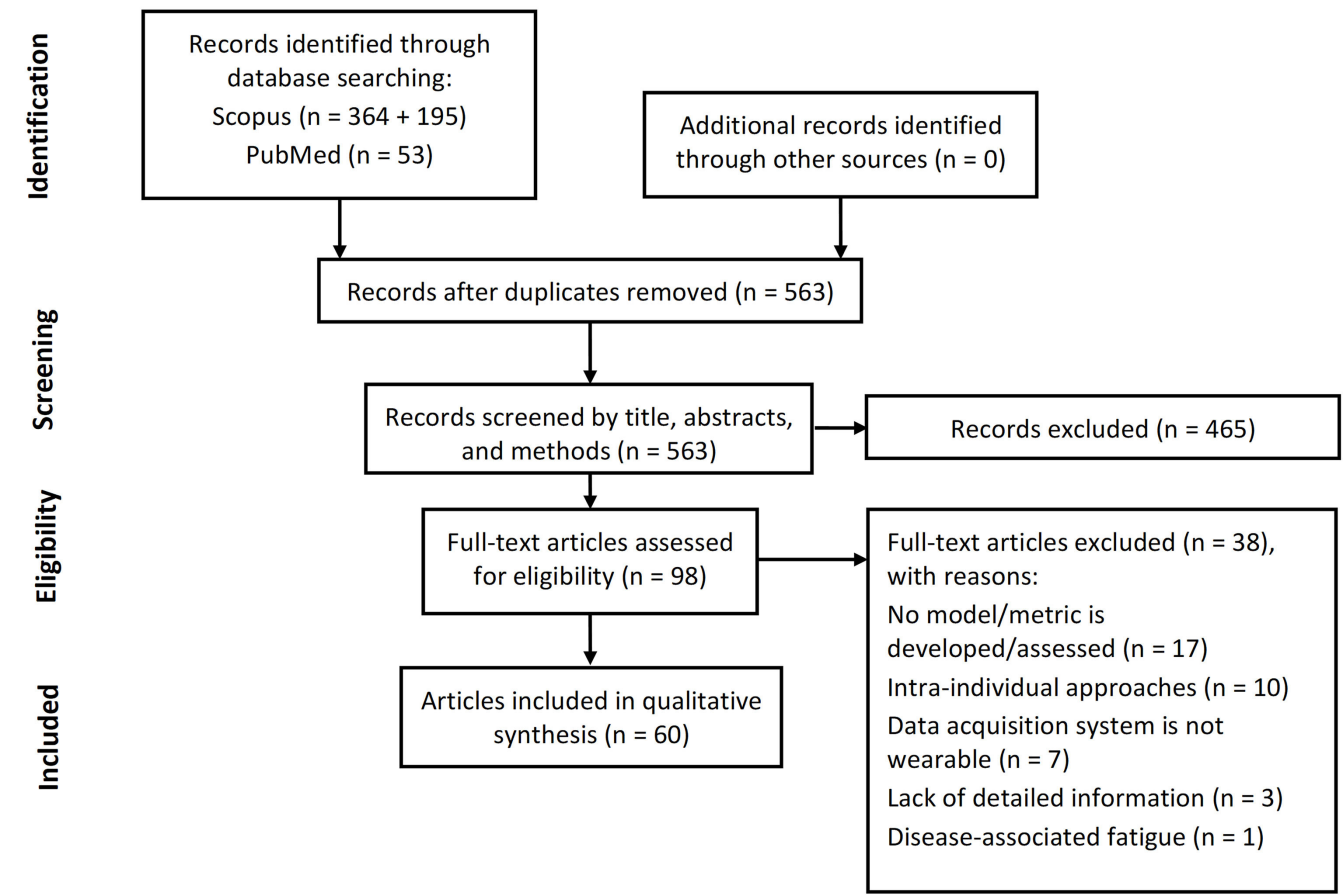
PRISMA flowchart. Flow of information through the different phases of articles selection process.

A total of 612 articles were retrieved in the literature search. After removing duplicates 563 remained. Of the identified articles, 465 were discarded during the screening phase due to lack of part of the inclusion criteria. The full text of the remaining 98 articles was assessed for eligibility. Of these, 38 were excluded for not developing a *fatigue* model/index or not assessing its performance (*n* = 17), proposing intra-individual approaches (*n* = 10), not using a wearable system to acquire data (*n* = 7), or lacking detailed information regarding the procedure used for model/index development (e.g., type of data used to develop the model, data acquisition system, etc., *n* = 3). Articles addressing disease-associated fatigue (Motta et al., 2016) were deemed to be outside the scope of this review and were therefore excluded (*n* = 1). Thus, 60 articles were included in this review: 26 conference proceedings and 34 journal articles. The articles selection process is summarized in Figure 1.

For maximum clarity, each article has been assigned a unique number by which it will be referred to throughout this work. Table 1 provides the list of included articles. Among them, eight address mental fatigue (#1–#8), three vigilance detection (#9–#11), 34 drowsiness (#7, #12–#44), 12 physical fatigue (#45–#56), and four muscle fatigue (#57–#60). A total of 59 different studies are included in the present review as article #46 acknowledged the use of previously collected data (from #53).

**Table 1:** List of included studies with the respective number and citation information, starting from studies addressing mental fatigue (#1–#8), followed by studies addressing vigilance detection (#9–#11), drowsiness (#12–#44), physical fatigue (#45–#56) and, lastly, muscle fatigue (#57–#60).

**No**.	**Citation**	**Title of Paper**
1	Zeng et al., 2020	Nonintrusive monitoring of mental fatigue status using epidermal electronic systems and machine-learning algorithms
2	Li et al., 2020	Identification and classification of construction equipment operators' mental fatigue using wearable eye-tracking technology
3	Lamti et al., 2019	Mental fatigue level detection based on event related and visual evoked potentials features fusion in virtual indoor environment
4	Zhang Y. et al., 2018	A deep temporal model for mental fatigue detection
5	Lee et al., 2019b	Emotion and fatigue monitoring using wearable devices
6	Huang et al., 2018	Detection of mental fatigue state with wearable ECG devices
7	Choi et al., 2018	Wearable device-based system to monitor a driver's stress, fatigue, and drowsiness
8	Al-Libawy et al., 2016	HRV-based operator fatigue analysis and classification using wearable sensors
9	Samima et al., 2019	Estimation and quantification of vigilance using ERPs and eye blink rate with a fuzzy model-based approach
10	Wang et al., 2019	Detecting and measuring construction workers' vigilance through hybrid kinematic-EEG signals
11	Chen et al., 2018	Developing construction workers' mental vigilance indicators through wavelet packet decomposition on EEG signals
12	Ko et al., 2020	Eyeblink recognition improves fatigue prediction from single-channel forehead EEG in a realistic sustained attention task
13	Sun et al., 2020	Recognition of fatigue driving based on steering operation using wearable smart watch
14	Foong et al., 2019	An iterative cross-subject negative-unlabeled learning algorithm for quantifying passive fatigue
15	Wen et al., 2019	Recognition of fatigue driving based on frequency features of wearable device data
16	Zhang M. et al., 2018	An application of particle swarm algorithms to optimize hidden markov models for driver fatigue identification
17	Zhang et al., 2017	Design of a fatigue detection system for high-speed trains based on driver vigilance using a wireless wearable EEG
18	Fu et al., 2016	Dynamic driver fatigue detection using hidden markov model in real driving condition
19	Boon-Leng et al., 2015	Mobile-based wearable-type of driver fatigue detection by GSR and EMG
20	Ko et al., 2015	Single channel wireless EEG device for real-time fatigue level detection
21	Kundinger and Riener, 2020	The potential of wrist-worn wearables for driver drowsiness detection: a feasibility analysis
22	Kundinger et al., 2020a	Assessment of the potential of wrist-worn wearable sensors for driver drowsiness detection
23	Kundinger et al., 2020b	Feasibility of smart wearables for driver drowsiness detection and its potential among different age groups
24	Gielen and Aerts, 2019	Feature extraction and evaluation for driver drowsiness detection based on thermoregulation
25	Mehreen et al., 2019	A hybrid scheme for drowsiness detection using wearable sensors
26	Kim and Shin, 2019	Utilizing HRV-derived respiration measures for driver drowsiness detection
27	Kartsch et al., 2019	Ultra low-power drowsiness detection system with BioWolf
28	Lee et al., 2019a	Using wearable ECG/PPG sensors for driver drowsiness detection based on distinguishable pattern of recurrence plots
29	Dhole et al., 2019	A novel helmet design and implementation for drowsiness and fall detection of workers on-site using EEG and random-forest classifier
30	Ogino and Mitsukura, 2018	Portable drowsiness detection through use of a prefrontal single-channel electroencephalogram
31	Nakamura et al., 2018	Automatic detection of drowsiness using in-ear EEG
32	Zhou et al., 2018	Vigilance detection method for high-speed rail using wireless wearable EEG collection technology based on low-rank matrix decomposition
33	Lemkaddem et al., 2018	Multi-modal driver drowsiness detection: a feasibility study
34	Li and Chung, 2018	Combined EEG-gyroscope-tDCS brain machine interface system for early management of driver drowsiness
35	Li et al., 2015	Smartwatch-based wearable EEG system for driver drowsiness detection
36	Li and Chung, 2015	A context-aware EEG headset system for early detection of driver drowsiness
37	Lee et al., 2016	Standalone wearable driver drowsiness detection system in a smartwatch
38	Leng et al., 2015	Wearable driver drowsiness detection system based on biomedical and motion sensors
39	Zhang S. et al., 2018	Low-power listen based driver drowsiness detection system using smartwatch
40	Cheon and Kang, 2017	Sensor-based driver condition recognition using support vector machine for the detection of driver drowsiness
41	Rohit et al., 2017	Real-time drowsiness detection using wearable, lightweight brain sensing headbands
42	Niwa et al., 2016	A wearable device for traffic safety - a study on estimating drowsiness with eyewear, JINS MEME
43	Ha and Yoo, 2016	A multimodal drowsiness monitoring ear-module system with closed-loop real-time alarm
44	Lee et al., 2015	Smartwatch-based driver alertness monitoring with wearable motion and physiological sensor
45	Sedighi Maman et al., 2020	A data analytic framework for physical fatigue management using wearable sensors
46	Nasirzadeh et al., 2020	Physical fatigue detection using entropy analysis of heart rate signals
47	Torres et al., 2020	Detection of fatigue on gait using accelerometer data and supervised machine learning
48	Khan et al., 2019	A novel method for classification of running fatigue using change-point segmentation
49	Ameli et al., 2019	Quantitative and non-invasive measurement of exercise-induced fatigue
50	Zhang et al., 2019b	Automated monitoring of physical fatigue using jerk
51	Tsao et al., 2019	Using non-invasive wearable sensors to estimate perceived fatigue level in manual material handling task
52	Wang et al., 2018	A heterogeneous ensemble learning voting method for fatigue detection in daily activities
53	Sedighi Maman et al., 2017	A data-driven approach to modeling physical fatigue in the workplace using wearable sensors
54	Aryal et al., 2017	Monitoring fatigue in construction workers using physiological measurements
55	Li et al., 2017	A neuro-fuzzy fatigue-tracking and classification system for wheelchair users
56	Buckley et al., 2017	Binary classification of running fatigue using a single inertial measurement unit
57	Karvekar et al., 2019	A data-driven model to identify fatigue level based on the motion data from a smartphone
58	Papakostas et al., 2019	Physical fatigue detection through EMG wearables and subjective user reports - a machine learning approach toward adaptive rehabilitation
59	Nourhan et al., 2017	Detection of muscle fatigue using wearable (MYO) surface electromyography based control device
60	Mokaya et al., 2016	Burnout: a wearable system for unobtrusive skeletal muscle fatigue estimation

Studies have examined the use of wearable technologies for *fatigue* quantification in occupational settings as well as for healthcare, sports, and exercise applications. Among these, *fatigue* in occupational settings, and in particular *fatigue* in transportation, has been the most extensively researched topic. In 44 of the 59 selected studies, authors propose systems for *fatigue* monitoring in occupational settings (Table 2). From the 44 studies targeting occupational settings, 29 introduce methods for *fatigue* detection in transportation, of which 27 address driver drowsiness monitoring (#7, #12–#16, #18–#24, #26, #28, #33–#44). High-speed train driver's drowsiness (#17, #32) has also been investigated.

**Table 2:** Application domain of wearable systems proposed in the included studies according to the concept they investigate.

**Type of *fatigue*/Application field**	**General-purpose**	**Healthcare**	**Sport and exercise**	**Transportation**	**Other occupations**	**Other applications**	**Total # studies**
Mental fatigue	#5	#4		#7	#1, #2, #6, #8	#3	8
Vigilance detection	#9				#10, #11		3
Drowsiness	#25, #30, #31			#7, #12–#24, #26, #28, #32–#44	#27, #29		34
Physical fatigue	#47	#52, #55	#48, #49, #56		#45, #46, #50, #51, #53, #54		11^*^
Muscle fatigue		#58	#60		#57, #59		4
Total # studies	6	4	4	29^*^	15^*^	1	59^*^

* *Article #7 addresses both drowsiness and mental fatigue; articles #46 and #53 use data from the same study*.

The application of wearable systems to monitor *fatigue* has been also foreseen in heavy industries such as construction (#2, #10, #11, #50, #54) and manufacturing (#45, #53) as well as in other occupations involving manual material handling tasks (#51). The prevention of work-related musculoskeletal disorders (#57, #59) along with overwork-related disorders (#6), and the monitoring of workers in mentally demanding occupations (#1) such as pilots and surgeons, are also among occupation-related applications for this type of technology.

**Figure 2:**
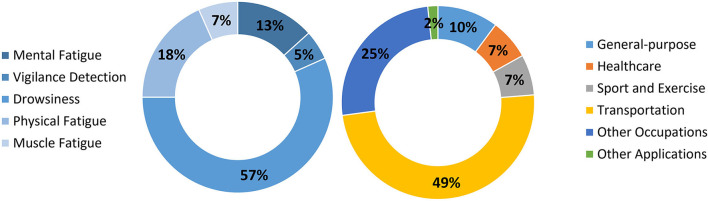
Type of *fatigue* and technology application domain.

The remaining *fatigue* monitoring studies were allocated to the other application domains such as sport and exercise (#48, #49, #56, #60), brain-computer interfaces (#3) as well as healthcare (#4). *Fatigue* monitoring in rehabilitation (#58), prevention of fatigue-induced falls and injuries (#52), and monitoring of manual wheelchair users' *fatigue* (#55) are some examples of healthcare-related applications. Figure 2 gives an overview of the proportion of research in wearable technologies for *fatigue* monitoring according to the type of *fatigue* and systems' application domains.

The current literature emphasizes the use of wearable systems to acquire subjects' physical and physiological data, which lays the foundation for *fatigue* quantification through behavioral- and physiological signal-based methods. Thus, the various approaches proposed for *fatigue* monitoring using wearable devices over the last five years fall within one of these two methods for *fatigue* quantification. A summary of study characteristics is given separately for each category of *fatigue* in Tables 3–**7**.

**Table 3:** Summary of characteristic of studies that investigated mental fatigue quantification using wearable devices.

**No**.	**Type of study**	**# of participants**	**Fatiguing task**	**Task duration**	**Input data**	**Reference measure**	**Modeling approach**	**Output**	**Model performance**
1	Lab	3	Mental arithmetic operations, reading professional literature	60 min	ECG, GSR, RES	Subjective self-report questionnaires, reaction time test	Decision Trees	3 levels	ACC >84%
2	Lab	6	Excavation operating simulation	60 min	Eye movement	SSS, NASA-TLX, task performance	SVM	3 levels	ACC = 85% precision = 86.6% recall = 85.9% *F*_1_ score = 85.1%
3	Lab	10	Virtual navigation	90 min	EEG	NASA-TLX	Dempster–Shafer fusion technique	4 Levels	*F*-score = 60–80%
4	Field	6	*Not stated*	*Not stated*	PPG, GSR, T_Sk_	CFQ	Deep convolutional autoencoding memory network	Binary	ACC = 82.9%
5	Lab	10	*Not stated*	*Not stated*	GSR, PPG	Likert scale	Multilayer neural networks	4 levels	ACC = 71.2%
6	Lab	29	Quiz with 55 questions	54 ± 8 min	ECG	CFQ	K-nearest neighbors	Binary	ACC = 75.5% AUC = 0.74
7	Lab	28	Simulated driving	150 min	GSR, PPG, T_Sk_, motion	*Not stated*	SVM	4 states (normal, stressed, fatigued and/or drowsy)	ACC = 68.31% (4 states)/ ACC = 84.46% (3 states)
8	Field	6	*Not stated*	*Not stated*	ECG, T_Sk_	HRV metric	SVM	Binary	ACC = 94.3%^*^

*ECG, electroencephalogram; EEG, electroencephalogram; GSR, galvanic skin response; PPG, photoplethysmogram; RES, respiration; T_Sk_, skin temperature; CFQ, the Chalder fatigue scale; HRV, heart rate variability; NASA-TLX, NASA task load index; SSS, Stanford sleepiness scale; SVM, support vector machine; ACC, accuracy; AUC, area under the receiver operating characteristic curve*.

* *Calculated average of classes (97.2% for alert, and 91.3% for fatigued state)*.

### Study Design

The vast majority of the studies were conducted in laboratory environments (*n* = 54). Studies involved 3–50 subjects, with an average of 14 participants. They focus particularly on short-term monitoring, however, three studies explored long-term *fatigue* monitoring in real-world settings (six weeks of monitoring in #4, 30 days in #8, and one week in #30). Although the use of selected tasks to induce *fatigue* was found to be prevalent (in 54 studies), none of the long-term studies reported the type of fatigue-inducing tasks subjects performed over the monitoring period.

### Fatigue-Inducing Tasks

Fatigue-inducing tasks varied greatly among studies, not only due to the type of *fatigue* investigated, but also in view of the system's final application (see Tables 3–**7**, columns “Fatiguing task” and “Task duration”). Among them, simulated tasks were found to be predominant, especially in studies exploring driver drowsiness (**Table 5**). Resemblance to real-world settings and avoidance of the risk associated with drowsy driving are the main reasons for this preference.

### References Measures of *fatigue*

The included articles used several reference measures of *fatigue*. Like fatigue-inducing tasks, gold-standard measures differed between studies, even among those investigating the same type of *fatigue* (see Tables 3–**7**, column “Reference measure”). Few studies did not use any reference measure for *fatigue* based on the assumption that subjects would be in a non-fatigued state before performing the fatigue-inducing task, and in a fatigued state right after the task. However, the use of reference measures of *fatigue* provides a means to confirm the success in inducing *fatigue*.

Reference measures used in the studies comprised of validated questionnaires and scales as well as other subjective assessments; behavioral-based measures, either based on direct observation by investigators or a physician, or based on video recordings; performance measures, including task performance and reaction time; and physiological-based measures derived from HR and HR variability (HRV), EEG, EMG as well as blood lactate levels.

Each of these *fatigue* measurement methods has its own limitations. In particular, subjective scales and questionnaires can give different outcomes for similar physiological changes between subjects. To cope with this limitation, Karvekar et al. (#57) used a short perception of the exertion calibration process to improve consistency among subjects.

### *Fatigue* Quantification Approaches

In most of the articles, the authors have proposed several *fatigue* models and compared their performance. Only the models considered to perform the best are presented in this review. From the included articles, 53 have proposed learning algorithms to model *fatigue* (see Tables 3–**7**, column “Modeling approach”). Within those learning algorithms, supervised machine learning approaches are prevalent, in particular support vector machines. Supervised learning consists of using input-output paired samples to infer the function that maps the input data (i.e., physiological and motion data) to the output (i.e., *fatigue* level). It relies on the availability of labeled data, and thus requires the use of a gold-standard measure of *fatigue*.

The labeling of datasets is usually done manually. However, Li et al. (#2) have proposed an automatic data labeling method based on Toeplitz Inverse Covariance-based Clustering (TICC). This clustering method identifies repeated patterns in multivariable time series and clusters time series subsequences accordingly, enabling automatic data labeling. In their work, 3 levels of mental fatigue were inferred based on TICC, namely low level, transition phase, and high level. Foong et al. (#14) have approached the problem of *fatigue* detection from a different perspective. They labeled a small amount of data acquired while subjects were in an alert state (positive class) and used features from these data to iteratively extract, from an unlabelled dataset, drowsy data (negative class). When using this algorithm, referred to as iterative negative-unlabelled learning algorithm, gold-standard values of subjects' *fatigue* level are no longer necessary. Foong argued that labeling alert data is easier and more precise than labeling *fatigue* states.

Most research has addressed *fatigue* quantification as a binary classification problem, meaning that proposed solutions determine the presence or absence of *fatigue* at a given point in time (see Tables 3–**7**, column “Output”). On the other hand, some researchers have addressed it as a multilevel problem (*n* = 13), considering three to five discrete levels of *fatigue*. Studies exploring both approaches (#21, #33, #34, #57) have found that the binary approach tends to result in better performance, even though reported improvement in accuracy ranges from <1 to 30%.

To differentiate *fatigue* in discrete levels, researchers have often set decision rules based on reference measure thresholding. It is of interest to note that studies using the same reference measure of *fatigue* still diverge on the measurement frequency and applied thresholds. As shown in **Table 8**, this was the case for the Karolinska Sleepiness Scale (KSS), the most used scale among studies investigating drowsiness (Table 5). Karolinska Sleepiness Scale was recorded at intervals of 1–10 min and subjects were deemed drowsy if their ratings were equal or higher than 5/9, 7/9, or 9/9, depending on the study. This divergence can also be observed in studies using Borg's RPE, a scale commonly used in studies exploring physical fatigue (Table 6).

**Table 4:** Summary of characteristic of studies that investigated vigilance detection quantification using wearable devices.

**No**.	**Type of study**	**# of participants**	**Fatiguing task**	**Task duration**	**Input data**	**Reference measure**	**Modeling approach**	**Output**	**Model performance**
9	Lab	10	Two-phase Mackworth Clock Test	30 min	EEG (brain activity and eye blinking)	Performance	Fuzzy logic	4 levels	ACC = 95%
10	Lab	10	Three-trial construction task (moving two mental tubes to a predefined location)	*Not stated*	EEG	NASA-TLX and EEG-vigilance stage model	Vigilance ratio index	Continuous vigilance level/3 levels	*r* = 0.73/*r* = 0.75
11	Lab	10	Three-trial construction task (moving two mental tubes to a predefined location)	*Not stated*	EEG	EEG-based benchmark	Vigilance ratio index	Continuous vigilance level	Cosine similarity = 0.90–0.92

*EEG, electroencephalogram; NASA-TLX, NASA Task Load Index; ACC, accuracy; r, correlation coefficient*.

**Table 5:** Summary of characteristic of studies that investigated drowsiness quantification using wearable devices.

**No**.	**Type of study**	**# of participants**	**Fatiguing task**	**Task duration**	**Input data**	**Reference measure**	**Modeling approach**	**Output**	**Model performance**
7	Lab	28	Simulated monotonous driving	120 min	GSR, PPG, T_Sk_, motion	Video-based reference	SVM	4 states (normal, stressed, fatigued and/or drowsy)	ACC = 68.31% (4 states)/ ACC = 84.46% (3 states)
12	Lab	15	Simulated night-time highway driving (lane-departure paradigm)	60 min	EEG (brain activity and eye blinking)	Reaction time	Multiple Linear regression	Binary	Se = 58% Sp = 73% ACC = 68%
13	Lab	10	Simulated highway driving	Not stated	Motion	Video-based reference, KSS	SVM	Binary	ACC = 83.3%
14	Lab	29	Target hitting game (alertness activity) and simulated driving	7 min + 60 min	EEG	KSS	Iterative negative-unlabelled learning algorithm	Subject's most fatigued block	ACC = 93.8%
15	Lab	10	Simulated driving	90 min	Motion	Video-based reference	SVM	Binary	ACC = 82.6%
16	Lab	20	Simulated driving	120 min	Eye movement	Real fatigue probability calculated based on heart rate test and subjective evaluation	HMM	Binary	ACC = 80%
17	Lab	10	Simulated train driving while sleep deprived	*Not stated*	EEG	Investigator's observation	SVM	Binary	ACC = 90.7% Se = 86.8% FP = 5.4%
18	Field	12	Real highway driving	210 min	EEG, EMG, RES, context	*Not stated*	First order HMM	Probabilities of fatigue	AUC = 0.84^*^
19	Lab	6	*Not stated*	*Not stated*	EMG, GSR	KSS, physician observation	SVM	Binary	Precision rate = 92%
20	Lab	15	Simulated driving	60 min	EEG	Reaction time	Linear regression model	Reaction time	ACC = 93.9% RMSE = 219.98 ms
21	Lab	28	Level-2 automated ride	45 min	PPG	KSS	KNN	Binary/3 levels	ACC = 99.4% *F*-score = 0.99/ ACC = 98.5% *F*-score = 0.99
22	Lab	27	Simulated automated ride	45 min	PPG	Weinbeer scale, micro-sleep events (based on eye closure duration)	Decision Stump	Binary	ACC = 73.4% *F*-score = 0.74^**^
23	Lab	10 (study A)/30 (study B)	Simulated monotonous driving under sleep deprivation/simulated monotonous driving	60 min/45 min	PPG	KSS and video-based reference/KSS	Subspace KNN	Binary	ACC = 99.9% Se = 100% Sp = 100% precision = 100% NPV = 100%
24	Lab	19	Simulated monotonous driving under different lightning conditions and levels of communication between subjects and researcher	90–150 min	T_Sk_	SSS	Decision Trees	Binary	Se = 77.8% Sp = 100% ACC = 88.9%
25	Lab	50	Watching a 3D rotating screen saver while sitting on a comfortable seat and sleep deprived	20 min	EEG (brain activity and eye blinking), motion	KSS	SVM with linear kernel	Binary	ACC = 86.5% (LOOCV)/ ACC = 92% Se = 88% Sp = 96% precision = 95.6% (Hold-out validation)
26	Lab	6	Simulated driving	60 min	ECG	Video-based reference	SVM regression	Binary	AUC = 0.95
27	Lab	3	Simulating drowsy state in the late night	5 times per state (approx. 4 min in total)	EEG (brain activity and eye blinking), motion	Parameters threshold determined by authors	Nearest Centroid Classifier based on K-means clustering	5 levels	ACC = 83%
28	Lab	6	Simulated driving	60–120 min	ECG/PPG	Video-based reference	Convolutional neural network	Binary	ACC = 70%/64% precision = 71%/71% recall = 85%/78% F-score = 77%/71%
29	Lab	4	Hand-eye-coordination game in a sleep deprived condition	approx. 8 min (500 s)	EEG	*Not stated*	Random forest	3 states (normal, sleepy, fallen)	ACC = 98%
30	Field	29	*Not stated*	*Not stated*	EEG	KSS	SVM with radial basis function kernel	Binary	precision = 73.5% Se = 88.7% Sp = 45.2% ACC = 72.7% F-score = 80.4%
31	Lab	23	4 naps while sleep deprived	20 min each nap	EEG	Clinician scoring (based on EEG data)	SVM with radial basis function kernel	Binary	ACC = 80% Kappa coefficient = 0.53
32	Lab	10	Simulated high-speed train driving while sleep deprived	*Not stated*	EEG	*Not stated*	Robust principal component analysis algorithm	Binary	ACC = 99.4%
33	Lab	15	Simulated driving	60 min	PPG	KSS, reaction time, total overrun area	KNN	Binary/3 levels	ACC = 93%/75%
34	Lab	17	Simulated monotonous driving	60 min	EEG, motion	Wierwille scale	linear SVM	Binary/5 levels	ACC = 96.2%/93.7%
35	Lab	20	Simulated monotonous driving	60 min	EEG	PERCLOS, number of adjustments on steering wheel	SVM-based posterior probabilistic model	3 levels (alert, early warning, full warning)	ACC = 89%^***^
36	Lab	6	Simulated monotonous driving	60 min	EEG, motion	Wierwille scale	SVM with linear kernel	Binary	ACC = 96.2% Se = 96.5% Sp = 95.6%
37	Lab	20	Simulated driving	60 min	Motion	KSS (rated by observer and confirmed by participant)	SVM	5 levels	ACC = 98.2%
38	Lab	20	Simulated driving	60 min	PPG, GSR, motion	KSS (rated by observer and confirmed by participant)	SVM	5 levels	ACC = 98.3%
39	Lab	4	Simulated driving	*Not stated*	PPG, motion	Video-based reference, driver's physical state	SVM with radial basis function kernel	Binary	ACC = 94.4% precision-recall score = 96.4%
40	Lab	10	Watching the photographed actual road video before and after doing a PVT	50 min	PPG	*Not stated*	SVM	Binary	ACC = 96.3% recall = 94.7% precision = 97.8%
41	Lab	23	Simulated driving while sleep deprived	60 min	EEG	*Not stated*	SVM with temporal aggregation	Binary	ACC = 87%
42	Lab	45	Sit on a chair and watch a movie of night driving while holding a steering wheel	80 min	EOG	Video-based reference	Random forest	Binary	ACC = 80%
43	Lab	*Unclear*	Sleep deprivation (4 h of sleep)	20 min	EEG, NIRS	Oxford Sleep Resistance Test	3^rd^ order polynomial SVM	3 levels (1, 2, or 3 consecutive missed stimulus)	ACC = 77.3%^****^
44	Lab	12	Simulated driving	480 min	PPG, motion	Observation, KSS (rated by observer)	Mobile-based SVM	Binary	ACC = 95.8%

*ECG,electroencephalogram; EEG,electroencephalogram; EMG,electromyogram; EOG,electrooculogram; GSR,galvanic skin response; NIRS,near-infra-red spectroscopy; PPG,photoplethysmogram; RES,respiration; T_Sk_,skin temperature; PVT,psychomotor vigilance test; KSS,Karolinska Sleepiness Scale; PERCLOS,percentage of eye closure; SSS,Stanford Sleepiness Scale; KNN,k-nearest neighbors; HMM,hidden Markov model; SVM,Support Vector Machine; ACC,accuracy; AUC,area under the receiver operating characteristic curve; FP,false positive; LOOCV,leave-one-out cross-validation; NPV,negative predictive value; RMSE,root mean square error; Se,sensitivity; Sp,specificity*.

* *Calculated average among all subjects (AUC between 0.734 and 0.960)*.

** *Calculated average of classes (0.82 for non-drowsy and 0.65 drowsy class)*.

*** *Average of the 3 states (91.25% for alert, 83.8% for early-warning group, and 91.9% for full warning group)*.

**** *Calculated average of classes (88.1 for 1, 77.9 for 2, and 65.9 for 3 missed stimulus)*.

**Table 6:** Summary of characteristic of studies that investigated physical fatigue quantification using wearable devices.

**No**.	**Type of study**	**# of participants**	**Fatiguing task**	**Task duration**	**Input data**	**Reference measure**	**Modeling approach**	**Output**	**Model performance**
45	Lab	15/13	Simulated manual material handling task/Supply pick-up and insertion task	180 min	Motion, age/ECG	Borg's RPE	Random forest	Binary	Se = 84.7/82.0% Sp = 86.4/88.9% ACC = 85.5/85.4% G-mean = 0.85/0.85 Consistency = 0.15/0.10
46	Lab	8	Part assembly task, supply pick-up, and insertion task, manual material handling task	180 min	ECG	Borg's RPE	Random Forest	Binary	ACC = 69.4–90.4% Se = 66.2–82.3% Sp = 71.7–96.2% AUC = 0.86–0.88
47	Lab	9	25 m shuttle sprint	Until sprint time decrement of 5% for two consecutive tests	Motion	*Not stated*	SVM	Binary	ACC = 90% Cohen's kappa = 0.75
48	Lab	12	Incremental treadmill running test	Time to exhaustion	EMG	Blood lactate samples	Random Forest	3 states (aerobic, anaerobic and recovery phase)	AUC = 0.86
49	Lab	20	Treadmill running program, L-drill and step test, crunch and jumps, sit to stand up, and push-up	7.5 min	Motion	Rate of perceived exertion a day after the protocol	Fatigue score	Binary	*r* = 0.95 (male) *r* = 0.70 (female)
50	Lab	6	Wall building/two bricklaying activities	Approx. 30 min/50 min	Motion	*Not stated*	SVM with quadratic kernel/SVM with medium gaussian kernel	Binary	ACC = 79.2%/ ACC = 65.9%
51	Lab	6	Lifting/lowering and turning task in 2 different paces (quick/slow)	5 min each task	RES, GSR, PPG	Borg's RPE	Linear regression model	3 levels of fatigue	Correct rate = 66.7% Absolute difference = 1.9 *R*^2^ = 0.39
52	Lab	15	Jumping rope consecutively	5 min (repeated until exhaustion)	Motion, age, height, weight	Maximum rope number	Heterogeneous ensemble learning voting method	Binary	ACC = 92% Precision = recall = = F1-score = 0.73
53	Lab	8	Simulated manufacturing tasks	180 min	ECG, motion	Borg's RPE	Least absolute shrinkage and selection operator model	Binary/RPE prediction	Se = 1, Sp = 0.79/ MAE = 2.16
54	Lab	12	Simulated construction activity	200 trials (approx. 150 min)	T_Sk_, ECG, personal information	Borg's RPE	Boosted trees	4 levels	ACC = 82.6%
55	Lab	8	Propel a wheelchair at a constant speed of 1.6 m/s	Until being unable to meet the required speed	ECG, EMG, motion	Self-reported fatigue	Neuro-fuzzy classifier	3 levels	ACC = 80%
56	Field	21	The Beep test or Pacer test until exhaustion	Time to exhaustion or Borg's RPE ≥18	Motion	Borg's RPE	Random Forest	Binary	ACC = 75% Se = 73% Sp = 77% F1-score = 75%

*ECG,electroencephalogram; EMG,electromyogram; GSR,galvanic skin response; PPG,photoplethysmogram; RES,respiration; T_Sk_,skin temperature; RPE,ratings of perceived exertion; SVM,Support Vector Machine; ACC,accuracy; AUC,area under the receiver operating characteristic curve; MAE,mean absolute error; r,correlation coefficient; R2, R-squared; Se, sensitivity; Sp, specificity*.

In #8, Al-Libawy et al. used individualized thresholds. Besides, Kim and Shin (#26) considered a subject to be fatigued from the first moment a drowsy event was detected until the end of the experiment. They assumed that subjects' drowsiness states will maintain if no action is taken against it.

Two studies (#30 and #53) have investigated the influence of the reference measure thresholds on the performance of supervised learning algorithms. Both studies have found that algorithms' performance alters according to the thresholds considered, and in #30 authors concluded that model performance improved when using the thresholds that maximize the distance between alert and drowsy states. Besides, #53 has shown that those thresholds can also alter the subset of predictors deemed relevant for *fatigue* level prediction.

As an alternative to classification, some studies have proposed solutions to monitor *fatigue* in a continuous manner, which does not require setting thresholds. This can be achieved through the prediction of *fatigue* indicators, e.g., reaction times or *fatigue* scale ratings, using regression models (see #20, #48, and #60). A Hidden Markov model (HMM, #16) and a vigilance index (#11) have also been proposed for the same purpose.

A few studies combined the continuous with the binary (#53) or multilevel (#10) approaches, instead of approaching the *fatigue* quantification problem from a single perspective. Two other studies proposed models to detect not only *fatigue*, but also other states strongly linked to safety and performance, for instance, stress and fall detection (#7, #29). Apart from learning algorithms, indices calculated based on the monitored parameters (#10, #11, #49) have also been developed for *fatigue* monitoring purposes. Furthermore, different statistical modeling techniques, namely Hidden Markov modeling, Dempster-Shafer Fusion technique, and Fuzzy logic, have been explored as described below.

The HMM describe the probability of transition between discrete, unobservable (hidden) states, based on observable parameters generated by those states (Wallisch et al., 2009). Such models have been widely applied for predicting sequences and time series due to their capability of modeling dynamic processes. This is the reason why #16 and #18 have developed HMM for *fatigue* monitoring.

Lamti el al. (#3) have proposed a Dempster-Shafer Fusion technique to determine subjects' *fatigue* levels. This technique allowed the fusion of features from data acquired by different sensors in a way that data uncertainties and heterogeneity were considered, thus providing a more robust estimation of *fatigue* levels.

Lastly, Samima et al. (#9) has proposed a fuzzy logic-based system for vigilance level monitoring. Fuzzy logic enables the numeric quantification of the different transitional levels between two opposite states, in this case, non-vigilance and vigilance. Samima's algorithm represents another approach to address *fatigue* as a continuous variable. Although the numerical values were grouped into four (no, more, moderate, or high) vigilance levels for the sake of model validation, the proposed model was found to be effective. A similar approach has been proposed by Li et al. (#35), who developed a support vector machine-based posterior probabilistic model which estimates driver drowsiness probability in values ranging from 0 to 1.

### Performance of Proposed Measures

The proposed approaches were found to perform very well in detecting *fatigue*, as seen in Tables 3–7, column “Model performance.” Reported accuracies range from approximately 70% to up to 100%. Despite such promising results, the performance of the proposed models on independent datasets has been assessed quantitatively in only one study. Kundinger et al. (#23) trained their models using different datasets which included data of young and/or old people. They evaluated models' performance in datasets other than the one used for model development and noticed that it results in lower accuracies, even when both datasets included data from people of the same age group. From the proposed approaches, four (#12, #27, #34, #35) were tested for real-time monitoring, revealing high accuracies for all approaches.

**Table 7:** Summary of characteristic of studies that investigated muscle fatigue quantification using wearable devices.

**No**.	**Type of study**	**# of participants**	**Fatiguing task**	**Task duration**	**Input data**	**Reference measure**	**Modeling approach**	**Output**	**Model performance**
57	Lab	24	2-min squatting at 8 squats/min	Time until Borg's RPE ≥17	Motion	Borg's RPE	Support vector machine	Binary/4 levels	ACC = 91/61%
58	Lab	10	Shoulder flexion, shoulder abduction, elbow extension performed using a Barrett WAM arm	Time until self-reported fatigue + 10 s (3 repetitions per exercise)	EMG	Self-report	Gradient Boosting	Binary	*F*_1_-score = 70.4–76.6% Success rate = 73–74%
59	Lab	3	Muscle fatiguing exercise	Time until self-reported fatigue	EMG	*Not stated*	Backpropagation neural networks	Binary	ACC = 100%
60	Field	5	Isotonic/isometric bicep curl, Isotonic/isometric leg extension	Time until failure (1–2 min)	Motion	Gradient of the Dimitrov spectral index (based on EMG)	Regression tree model	Gradient of the relative change in the Dimitrov spectral fatigue index	Error <15%

*EMG,electromyogram; RPE,ratings of perceived exertion; ACC,accuracy*.

### *Fatigue* Monitoring Systems

**Figure 3:**
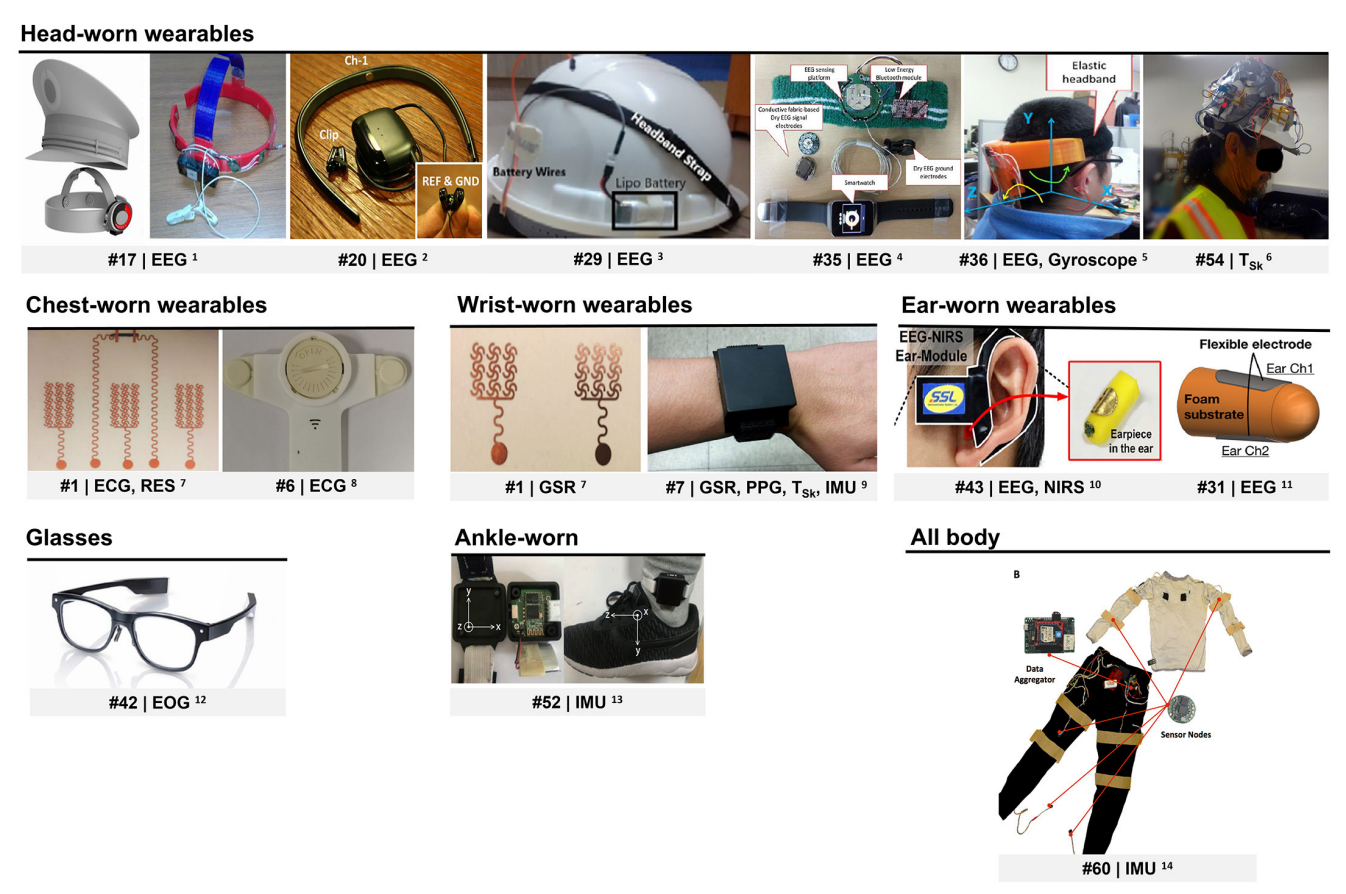
Wearables devised for *fatigue* monitoring. ECG, electrocardiogram; EEG, electroencephalogram; EOG, electrooculogram; GSR, galvanic skin response; IMU, inertial motion unit; NIRS, near-infra-red spectroscopy; PPG, photoplethysmography; RES, respiration; T_Sk_, skin temperature. ^1^Reprinted from Zhang et al. (2017). ^2^© (2015) IEEE. Reprinted, with permission, from Ko et al. (2015). ^3^Reprinted from Dhole et al., (2019). © (2019) with permission from Elsevier. ^4^© (2015) IEEE. Reprinted, with permission, from Li et al. (2015). ^5^Reprinted from Li and Chung (2015). ^6^Reprinted from Aryal et al. (2017). © (2017) with permission from Elsevier. ^7^Reprinted (adapted) with permission from Zeng et al. (2020). © (2020) American Chemical Society. ^8^Reprinted from Huang et al. (2018). © (2018), with permission from Elsevier. ^9^© (2018) IEEE. Reprinted, with permission, from Choi et al. (2018). ^10^© (2016) IEEE. Reprinted, with permission, from Ha and Yoo, (2016). ^11^© (2018) IEEE. Reprinted, with permission, from Nakamura et al. (2018). ^12^Republished with permission of SAE International, from Niwa et al. (2016); permission conveyed through Copyright Clearance Center, Inc. ^13^Reprinted with permission of Fuji Technology Press Ltd., from (Wang et al., 2018). ^14^© (2016) IEEE. Reprinted, with permission, from Mokaya et al. (2016).

Several wearable devices have been used to obtain data regarding subjects' *fatigue* level. Researchers have either employed wearable sensors available on the market or devised their own systems (*n* = 22, see Supplementary Table 4, column “Data acquisition system”). Figure 3 presents some of the devised wearable devices.

Motion (MOT) as measured by inertial measurement units (IMUs) and other motion sensors, EEG, photoplethysmography (PPG), electrocardiogram (ECG), galvanic skin response (GSR), and EMG are among the most investigated signals for *fatigue* level estimation. While less extensively studied, skin temperature (T_sk_), respiration (RES), EYE, including oculometry and pupillometry, electrooculogram (EOG), as well as near infra-red spectroscopy (NIRS) were also found to be relevant for *fatigue* detection.

**Figure 4:**
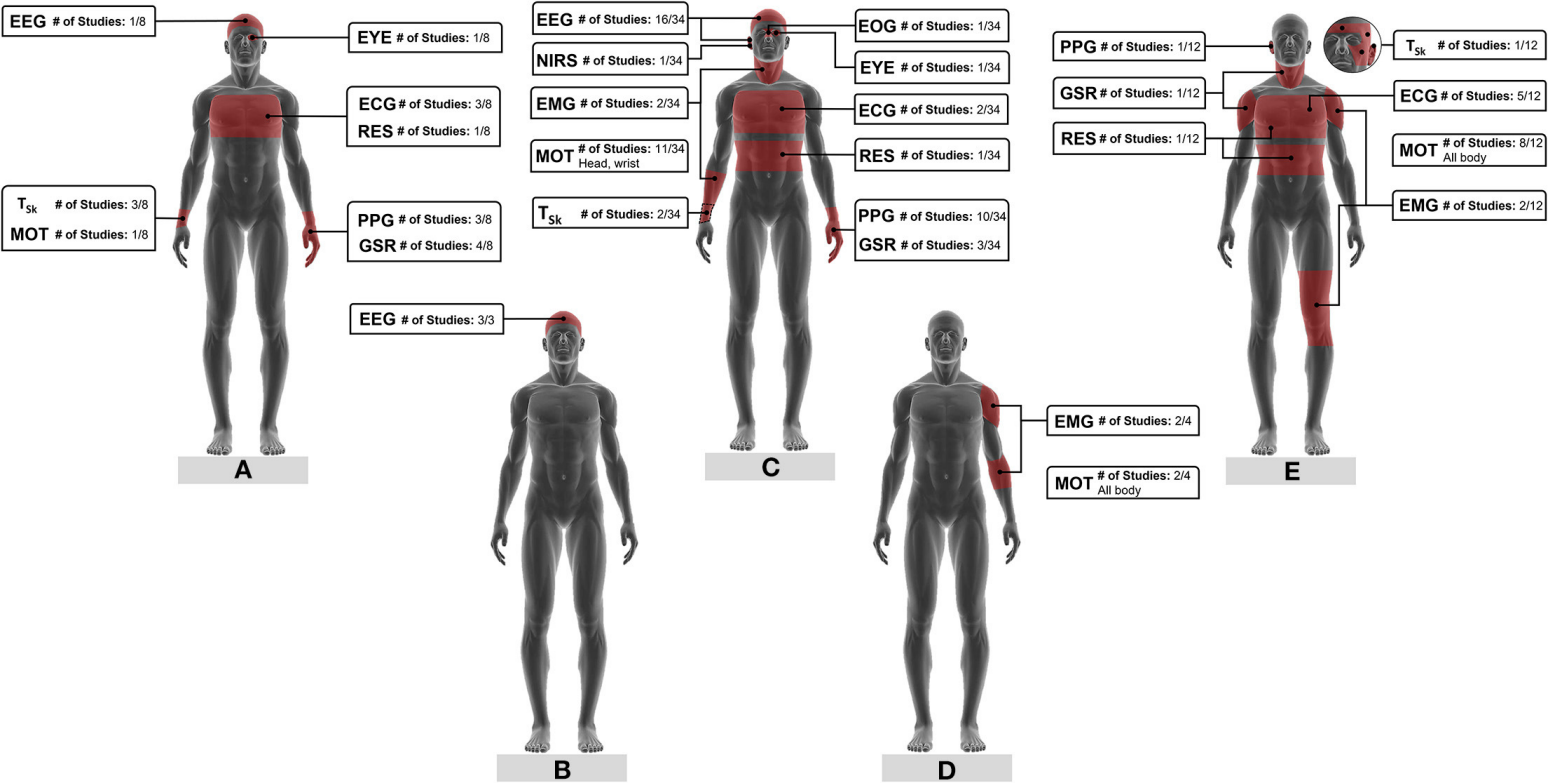
Physiological and motion signals for **(A)** mental fatigue, **(B)** vigilance, **(C)** drowsiness, **(D)** muscle fatigue, **(E)** physical fatigue monitoring and respective measurement locations. The fraction number in the boxes represents the number of studies on a specific signal based on the total literature reviewed on that type of *fatigue*. ECG, electroencephalogram; EEG, electroencephalogram; EMG, electromyogram; EOG, electrooculogram; EYE, eye movement; GSR, galvanic skin response; MOT, motion; NIRS, near-infra-red spectroscopy; PPG, photoplethysmogram; RES, respiration; T_Sk_, skin temperature.

Signals as well as respective measurement locations varied with the type of *fatigue* investigated but were rather consistent among different studies. Figure 4 shows the physiological and motion signals used to monitor the different types of *fatigue*, their measurement location, and the number of studies that have explored those signals.

Electroencephalogram is the most prominent signal for drowsiness monitoring (see Figure 4C; Table 5, column “Input data”). Furthermore, all the three studies investigating vigilance monitoring developed models and indices based on EEG data (see Figure 4B; Table 4, column “Input data”). Spectral power changes in the different frequency bands (#10–#12, #14, #17, #18, #20, #25, #27, #30, #32, #34 to #36, #41, #43) and event-related potentials (#3, #9) of EEG signals collected by wearable EEG systems contain valuable information on subjects' drowsiness, vigilance, and mental fatigue levels.

Almost all studies acquired EEG data from headbands (Supplementary Table 4, column “Data acquisition system”). Electroencephalogramelectrodes were placed on several brain regions, particularly on frontal (#12, #14, #29, #30) and occipital lobes (#18, #34, #35, #36). Researchers designed headbands with mainly two to three dry (#20, #27, #34, #35, #36) or wet (#29) electrodes, although systems with more channels were also proposed. Two studies presented an eight-channel EEG system with stainless steel (#17) and Ag–AgCl (#32) dry electrodes embedded in a train driver's cap (Figure 3). Besides, Dhole et al. (#29) integrated their EEG monitoring system into a safety helmet (Figure 3) which houses an IMU and a wireless transmission setup. Motion sensors were likewise included in the systems proposed by three other studies (IMU in #27, three-axis gyroscope in #34 and #36).

Nakamura et al. (#31) and Ha and Yoo (#43) devised wearable systems for in-ear EEG monitoring (Figure 3). The first system consists of a wearable in-ear sensor made of viscoelastic foam with two flexible cloth EEG electrodes constructed out of conductive fabric. Nakamura and coworkers showed that a drowsiness classification model trained on their system's data performs almost as well as a model developed based on a scalp EEG headset (average difference in accuracy of approximately 6%). The second system, in turn, monitors EEG and NIRS simultaneously. It is composed of an earpiece with two fabric electrodes for EEG monitoring and NIRS driver, and an ear hook for NIRS monitoring. Ha and Yoo's work revealed that oxyhemoglobin concentration in the brain, measured by in-ear NIRS, also provides information on human drowsiness. Their findings also indicate that the use of in-ear EEG in combination with in-ear NIRS improves drowsiness detection accuracy.

Several studies found that pulse intervals, HR, and HRV derived from PPG signals collected by wearables are relevant predictors of drowsiness (Figure 4C) and mental fatigue (Figure 4A). In those studies, PPG data was acquired from subjects' wrists or fingers using mainly commercial devices, although some employed self-made systems (#5, #7, #38, #44). Choi et al. (#7) designed a multi-sensor wrist band with PPG and T_sk_ sensors, GSR electrodes, as well as motion sensors (acceleration and rate of rotation) to monitor driving stress, fatigue, and drowsiness (Figure 3).

Studies also showed that wearable ECG sensors can be applied for *fatigue* detection. ECG signals were acquired by chest strap HR monitors, except in studies #1 and #6. Zeng et al. (#1) designed epidermal electronic sensors. They fabricated filamentary serpentine mesh electrodes made of copper and a graphite-based strain sensor for simultaneous ECG, GSR, and RES monitoring (Figure 3). These sensors are applied on the skin in the form of temporary tattoos. Huang et al. (#6) used a single-channel ECG patch to acquire the data (Figure 3).

Physical and mental fatigue were found to significantly affect subjects' HR, HRV, RR intervals as well as respective spectral power and dynamics. Lee et al. (#28) investigated the use of different types of recurrence plots to detect drowsiness. Those plots were constructed using pulse intervals from PPG or RR intervals. Their results suggest that recurrence patterns, known for capturing non-linear dynamics of HRV, are reliable predictors of drowsiness.

Wearable systems with GSR electrodes located at the wrist (#4, #7), hand palms (#1), fingers (#5, #19, #38), neck, and shoulders (#51) were exploited to monitor drowsiness, mental, and physical fatigue. GSR attributes such as its average, standard deviation, number of peaks, their magnitude, and duration, as well as information regarding signal frequency spectrum were used as predictors of *fatigue*. Skin conductance variations also served as indicators of drivers' stress (#7, #38).

The few studies exploring EMG signals showed that wearable EMG sensors could successfully monitor physical and muscle fatigue based on a single variable. Electromyogram signals combined with other variables (e.g., EEG, RES, GSR) also detects drowsiness. Data were acquired from arm (#19, #55, #58, #59), leg (#48), and neck (#18) muscles. While some studies collected EMG signals using conventional electrodes, wireless, dry electrodes (#48, #58, #59) were also applied.

Average T_sk_, its standard deviation (#4, #7, #8), slope (#24), and power spectral density (#8), have been shown to provide information on mental fatigue and drowsiness levels. All these studies measured T_sk_ using wrist-worn sensors. Aryal et al. (#54) took a different approach by estimating perceived physical fatigue from thermoregulatory changes monitored by temperature sensors on the face. T_sk_ data were acquired from subjects' temple, forehead, cheek, and ear using non-contact infra-red temperature sensors fitted into a construction safety helmet (Figure 3). Their results suggest that T_sk_, especially measured at the temple, can be more useful to model physical fatigue than heart rate data.

In addition to the physiological signals already mentioned, RES, EYE, and EOG have been investigated. Breathing rate (#1, #51), peak voltage (#51), and mean frequency power (#18) of RES signals were derived from wearable sensors placed on chest and/or abdomen area. These features can be combined with other physiological signals to estimate individuals' drowsiness and mental and physical fatigue levels.

Eye movement-related features extracted from eye-tracking headsets (#2, #16) were applied for mental fatigue and drowsiness detection. Niwa et al. (#42) extracted such information from a glasses-like wearable EOG device (see Figure 3). Moreover, studies #9, #12, #25, and #27, used blink-induced artifacts that contaminate EEG recordings to derive information regarding blink rate, amplitude, and duration. This approach was found to improve vigilance and drowsiness level prediction without requiring additional equipment.

Motion tracking has a preponderant role in physical and muscle fatigue as well as drowsiness detection. While whole-body motion was recorded for physical and muscle fatigue (Figures 4D,E), head and wrist movements seem to be the focus for drowsiness detection (Figure 4C). Information relating to body motion, posture, gait patterns, and drivers' steering behaviors (#13, #44) have been estimated from wearable inertial sensors. Kinetic energy, a measure of the energy required to conduct motions (#49); jerk, which is the time derivate of acceleration signals (#50, #53); joint angles (#49); Euler angles (#56) are some of the researched features.

Among the systems used to acquire motion data are mobile phones (#47, # 57), smart watches (#13, #15, #37, #38, #39, #44), head (#25, #27, #34, #36), and ankle bands (#52, Figure 3). Mokaya et al. (#60) developed Burnout, a wearable sensor network comprised of 10 lightweight sensor nodes embedded in fitted clothing (see Figure 3). Each sensor node contained a 3-axis accelerometer able to sense muscle vibration and detect body movement.

To cope with the inter-subject variability in *fatigue* development and its impact on individuals' motion and physiological signals, some studies (#2, #7, #52) normalized the acquired data prior to model construction. In Choi's work (#7), four methods of normalization were used. The normalization method was determined for each feature as the one that maximizes the separability between classes.

The use of demographic attributes, such as age, height, weight (#45, #52); contextual information, for instance, sleep quality, circadian rhythm, and work condition (#18); as well as the duration of work and years of working experience (#54), in combination with physiological and motion parameters, has been shown to enhance *fatigue* models performance. Aryal et al. (#54) found that the use of personal information improved their model accuracy by 15%.

Maman's study (#45) has shown that the relevance of different *fatigue* predictors varies with the task being performed. In their work, the same methodology was applied to data acquired while subjects were performing different manufacturing tasks. Maman et al. found that the features remaining after the selection process was different for the two tasks investigated.

### Risk of Bias Assessment

**Figure 5:**
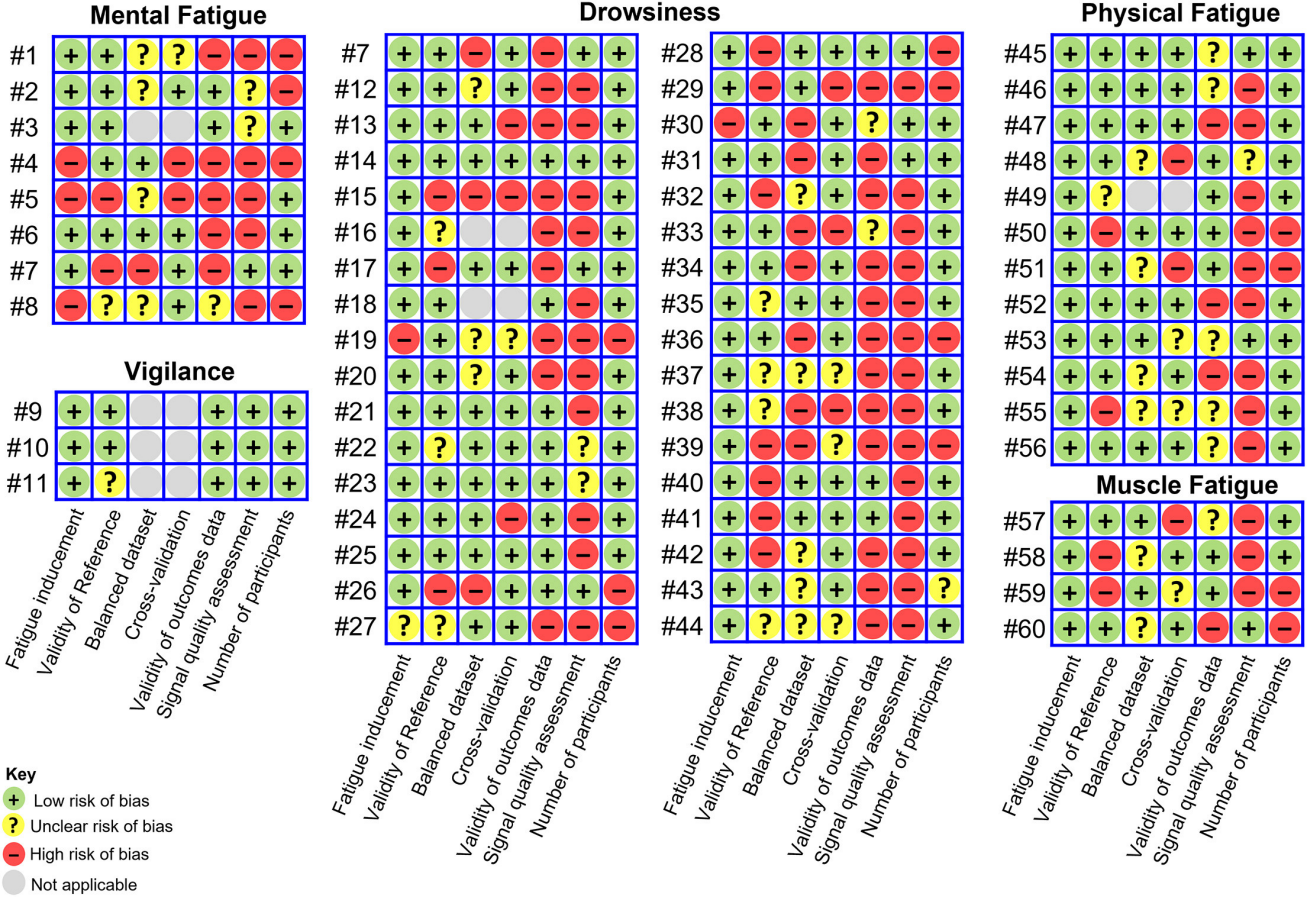
Risk of bias assessment. Studies with low risk of bias in all components were deemed to be of low risk of bias, studies with low or unclear risk of bias for all components were deemed to be of unclear risk and those with high risk of bias for one or more components were deemed to be of high risk of bias.

The risk of bias of included studies is depicted in Figure 5. Only few studies fell within the low (#9, #10, #14) or unclear risk of bias (#3, #11, #22, #23, #45, #53) categories. All the remaining studies were assigned to the high risk of bias category according to the criteria described in the Methods section.

Signal quality assessment was found to be the most neglected of the methodological aspects contemplated in the risk of bias assessment. Noise and artifacts arising from several sources have an influence on data quality and wearable systems are particularly susceptible to motion artifacts (Mihajlovic et al., 2015; Boudreaux et al., 2018; Choksatchawathi et al., 2020). While these facts are well known, only few studies (*n* = 8) identified low-quality signal segments with the purpose of excluding those segments from further analysis or reconstructing them. Some researchers assessed the data visually and manually removed artifacts (#45, #53, #60), while others used automatic approaches.

The automatic approaches consisted of feasibility rules applied through thresholding or combination of decision rules. Those rules were either based on changes in signal amplitude and shape (#14, #31) or on the physiological feasibility of parameters derived from the signal as compared to reference values in healthy subjects (#26, #28). Choi et al. (#7) applied a sequence of four decision rules to identify valid data segments from the acquired PPG signal. Their algorithm detected irregularities in the measured pulse intervals due to false peaks, evaluated the similarity between consecutive pulses, and assessed the amount of device motion during data acquisition. Choi reported in their article an average improvement of more than 7% in pulse detection after applying the algorithm. In their work, low-quality signal segments were removed without replacement. Conversely, one study used cubic interpolation (#26) and another the neighbors mean (#28) to reconstruct the signal after the exclusion of noisy data points.

Instead of assessing the quality of the acquired data, some studies applied more sophisticated noise removal algorithms and signal reconstruction techniques to ensure high data quality. Independent component analysis (#9) was to decompose EEG signals and reconstruct signals without artifacts by removing the artifactual components. Wavelet decomposition was also investigated for the same purpose (#17, #30). In studies #10 and #11, principal component analysis was used to select the most effective EEG channels, which were 14 in total. Signals from the selected channels were then averaged to remove artifacts.

In *fatigue* studies, the amount of data acquired while subjects are in a non-fatigued state tends to exceed the amount of data acquired while in a fatigued state. Since *fatigue* is the concept of interest, this imbalance needs to be addressed and was for that reason included in the risk of bias assessment. To avoid the creation of imbalanced datasets in the first place, some researchers have acquired data while subjects were rested and fatigued, following a similar methodology. However, imbalances can be corrected through resampling the data.

Maman (#53) explored the use of random under-sampling (removes cases from the majority class) and synthetic minority over-sampling (generates synthetic examples of the minority class by interpolation; Kotsiantis et al., 2006) to equalize the amount of data acquired in a non-fatigued and fatigued states. In their work, the use of random under-sampling resulted in better performance. Synthetic minority over-sampling was also used by Kundinger et al. (#21, #22), while Wang (#52) explored oversampling (duplicates cases from the minority class).

It is also possible to address the dataset imbalance problem at the algorithm level. Zhang (#4) constructed a one-class classifier, named Deep Convolutional Autoencoding Memory network, whose data training process is only based on non-fatigue data. This model learns patterns in time series of non-fatigue data and classifies outliers as data recorded under *fatigue* state. More detailed information on the risk of bias assessment of the included studies can be found in Supplementary Materials.

## Discussion

This review aimed at presenting the state-of-the-art of *fatigue* monitoring through wearable devices. A considerable amount of the current literature on the selected topic pays particular attention to drowsiness monitoring for occupational purposes, especially within the transportation industry (Figure 2). Other potential application domains foreseen for such kind of technology stretch across healthcare and sport industries (Table 2).

Overall, *fatigue* studies have been mainly short-term studies, conducted in laboratory settings. The tasks used to induce *fatigue*, selected reference measures and other methodological aspects differed considerably between studies. Researchers have used wearables available in the market or devised their own systems to acquire physiological and motion data and, based on these, detect *fatigue*. Supervised machine learning models, and more specifically, binary classification models, are predominant among the proposed *fatigue* quantification approaches.

Considering that wearables enable continuous, unobtrusive long-term monitoring, it is surprising that most of the articles only reported short-term laboratory studies. A possible explanation for this finding is shown by the main limitation of the three studies exploring long-term monitoring of *fatigue* in free-living environments (#4, #8, #30). It is the lack of information regarding the tasks performed by the participants over the monitoring period. As shown in Maman's study (#45), *fatigue* development is task-dependent (Richter et al., 2005; Yung and Wells, 2017), therefore, information regarding fatigue-inducing tasks plays a pivotal role in determining the validity domain of developed models.

Additionally, in real-world settings the acquisition of reference values of *fatigue* is further aggravated, particularly when using scales and questionnaires. Obtaining reference values in intervals of 2 h or three times a day, as applied in studies #4 and #30, might be inappropriate when aiming at developing a *fatigue* monitoring system for safety-related applications, for instance. At the same time, there is a lack of valid reference measures which can provide a continuous estimate of an individual's *fatigue* level non-intrusively. Hence, considerations regarding fatigue-inducing tasks and reference measures to be used are a major challenge in *fatigue* research.

Most of the included studies used fatigue-inducing tasks. Those tasks varied markedly from study to study, depending on the type of *fatigue* investigated and technology final application. Simulated tasks have been commonly used, particularly in studies investigating driver drowsiness. Despite resembling real settings, simulation environments cannot reproduce its complexity and dynamism (Techera et al., 2018). Additionally, subjects' effort and perception of risk are reduced in simulated environments (Sahayadhas et al., 2012). The effectiveness of the tasks used to induce *fatigue* is key for *fatigue* research in laboratory settings and depends to some extent on participants' engagement in those tasks. For this reason, Huang et al. (#6) offered monetary rewards to better motivate the participants. In general, the selection of fatigue-inducing tasks and their duration lacked scientific basis. Thus, questions can be raised regarding the feasibility of certain tasks which have been used.

Reference measures of *fatigue* also differed among studies. Some researchers neglected the use of ground truth measures of *fatigue* relying on the assumption that participants would be rested before performing the fatigue-inducing task and fatigued after that. Foong et al. (#14) questioned this assumption with regard to drowsiness. In their work, participants performed a hitting game prior to the fatigue-inducing task to ensure subjects were in an alert state at the start of the experiment. As noted by Ko et al. (#12), drowsiness does not evolve linearly in time, and the same can be stated with regard to *fatigue* in general. Indeed, there is a time-on-task effect on *fatigue* (Richter et al., 2005). Yet, for reasons unrelated to the study protocol, subjects may either be fatigued before performing the fatigue-inducing task or they may not become fatigued in case of high-performing individuals or if the selected task is not effective in inducing *fatigue*. For instance, Aryal et al. (#54) recognized, based on reference measures, that the selected fatiguing task failed to induce mental fatigue and consequently limited the scope of their investigation to physical fatigue. Therefore, the premise that subjects are rested/fatigued prior to/after the fatigue-inducing task might be a flawed one and does not justify neglecting the use of reference measures of *fatigue*.

Much of the literature has addressed *fatigue* monitoring as a binary classification problem. Considering that the major application foreseen for such a technology is *fatigue* risk management in occupational settings, detecting the occurrence of *fatigue* is not as relevant as detecting the transition to such a state (Li et al., 2020). That transition phase is crucial to prevent fatigue-related accidents since it is when corrective actions must be taken. Therefore, although applying reference measure thresholds which maximize the separability between rested and fatigued states might result in better model performance, it limits models' ability to detect the early onset of *fatigue*. In this sense, multilevel or continuous approaches might better address *fatigue* monitoring. However, the number of studies proposing such approaches is relatively low (*n* = 23).

Regardless the approach followed to address *fatigue* monitoring, the selection of the ground truth measure of *fatigue* and respective thresholds is crucial. It dictates the standard by which proposed models will be developed and assessed. The observed divergence between the reference measure thresholds applied in the included studies, even among those investigating the same type of *fatigue* for the same application (e.g., papers addressing driver drowsiness monitoring which used the KSS, see Table 8), reflects a lack of consensus regarding the adequate threshold to consider a given *fatigue* level as risky or unsafe. This divergence may be due to the lack of evidence on the association between a given *fatigue* level, task performance, and the related safety outcomes (Williamson et al., 2011).

**Table 8:** Subjective scales used in the different studies: measurement frequency and *fatigue* thresholds.

**Reference measure**	**No**.	**Measure frequency**	**Measure threshold**
**Karolinska sleepiness scale**	14	10 min	Not stated
	19	3 min	Not stated
	25	1 min	Not stated
	13	10 min	Alert: ≤3; Fatigue: ≥7
	21	5 min	Alert: ≤4; Fatigue: ≥5
	23	5 or 10 min	Alert: ≤4; Fatigue: ≥7
	30	3 times/day	Alert: <3; Fatigue: >7
	33	5 min	Alert: ≤8; Fatigue: = 9
	18	Not stated	Alert: <3; Transition: 3–5; Fatigue: 5–7
	22	5 min	Alert: ≤4; Transition: 5–6; Fatigue: ≥7
	33	5 min	Alert: ≤7; Transition: = 8; Fatigue: = 9
	37	Unclear	Level 1: 1–2; Level 2: 3–4; Level 3: 5–6; Level 4: 7–8; Level 5: 9
	38	Unclear	Level 1: 1–2; Level 2: 3–4; Level 3: 5–6; Level 4: 7–8; Level 5: 9
**Borg's rating of perceived exertion**	45	10 min	13
	46	1 h	15
	53	10 min	15
	56	Unclear	18
	58	After each fatiguing task set	No fatigue: <7; Fatigue: ≥15
	51	1 min	Low: ≤9.5; Medium: 9.5–13.5; High: 13.5
	54	Unclear	Low: 6–11; Medium: 12–14; High: 15–16; Very high ≥17
	58	After each fatiguing task set	No: <7; Low: ≥7; Medium: ≥11; High: ≥15

Models considering *fatigue* accumulation and recovery are lacking. Only few studies considered the temporal variation of *fatigue* predictors. *Fatigue* development is a dynamic process, and individuals' *fatigue* level at a given point in time is not independent from their state at previous time points. Therefore, as noted by Fu (#18), the assumption of independent and identically distributed data, commonly applied in learning algorithms, is a poor one in the case of *fatigue*.

Despite their constraints, the performance of the proposed models was very high. Binary classification models performed particularly well. Yet, this promising set of results should be interpreted with caution as most of the models have not been tested in independent datasets.

Furthermore, previous studies have given little attention to the data imbalance problem. Although some studies report the number of data samples used to train the models, half of the studies using learning algorithms either omitted that information or acknowledged the use of imbalanced datasets (Figure 5). Just a few researchers reported strategies to cope with class imbalance. Thus, there is the risk that models were developed using imbalanced datasets, which implies that reported accuracies may overestimate models' performance, especially classification models performance (Luque et al., 2019).

In some works, data have been acquired prior to and right after subjects performed the fatiguing task. While being a useful method to ensure the creation of balanced datasets, the acquired data does not contain information regarding *fatigue* mechanisms. Consequently, models developed using those datasets can be relevant to analyze which and how the monitored variables change due to *fatigue* but have limited applicability in domains where continuous, long-term monitoring is necessary.

A third aspect weakening the reliability of some of the proposed models is the very little effort made to ensure the acquisition of high-fidelity signals. Only 21 studies used exclusively validated data acquisition systems, and even fewer removed artifacts or noisy signal segments (Figure 5). The exclusion of low-quality data is of particular importance because it is well known that wearable devices are susceptible to noise and artifacts arising from several factors, especially sensors' movement (Shirmohammadi et al., 2016). Thus, most of the proposed *fatigue* models and indices were constructed based on data of limited reliability.

We found that a wide variety of physiological and motion signals have been recorded for the purpose of *fatigue* quantification. Signals' measurement location did not differ considerably, but their relevance and pre-eminence for *fatigue* monitoring depended on the type of *fatigue* explored (Figure 4). Even though several signals have been monitored, there has been little attempt to understand how those signals change with *fatigue* onset and which data features better represent it. We believe this explains the great variability of features derived from the monitored signals. The task dependency of *fatigue* development and its impact on subjects' physiological and motion signals further contributes to that variability.

Together, the findings of this review reveal as much about the complexity involved in *fatigue* monitoring using wearable sensors as about the lack of standardization in this field of research. To ensure generalization to real-world settings, proposed approaches must be developed based on data acquired under realistic conditions. At the same time, models construction demands more controlled conditions. In addition, *fatigue* development is a dynamic process and its impact on subjects' performance depends on the task being performed. Such requirements demand research conducted in close collaboration with industry, the end user of developed technologies.

Researchers should be aware of current issues highlighted in the literature. Accordingly, future studies aiming at developing reliable wearable *fatigue* monitoring systems should consider the following methodological aspects:

1. When designing a study, care should be exercised when selecting the fatiguing tasks as well as their duration and workload. They should resemble the target application context and be well-founded on existing evidence.
2. The validity and reliability of the data acquisition systems should be assessed prior to recording the data for model construction. Acquired data should be pre-processed to correct recoverable disturbances and, afterwards, analyzed using a signal quality assessment algorithm to detect remaining low-quality signal segments and outliers (Naseri and Homaeinezhad, 2015). This approach allows the evaluation of the effectiveness of techniques applied to reconstruct acquired signals.
3. It is important to use validated reference measures of *fatigue*. In their review, Hu et al. (Hu and Lodewijks, 2020) advise the use of more than one reference measure of *fatigue* to cope with the multidimensionality of this concept. Another advantage of using several reference measures is that it allows the evaluation of the agreement between the type of *fatigue* induced by selected fatiguing tasks and the one of interest.
4. Techniques to handle data imbalances and cross-validation need to be implemented, if applicable. It allows the unbiased estimation of models' performance. Besides, the amount of data samples used for model development should be clearly reported.
5. The etiology of *fatigue* is multifactorial, and physiological and motion responses reflect the integration of various factors. Therefore, the impact of confounding factors (e.g., emotional state, health condition, or even other types of *fatigue* apart from the one of interest, etc.) should be considered and assessed.

The potential of wearables for *fatigue* monitoring has not yet been explored fully. Long-term studies, in which *fatigue* is monitored continuously in real-world environments are lacking. However, for such applications, preceding studies about the development of reliable models in laboratory settings and the subsequent implementation in free-living environments are needed. This enables model validation and, at the same time, provides new insights into the impact of *fatigue* on individuals' performance and related outcomes.

Long-term studies would also allow the investigation of *fatigue* dynamics. Given the intra- and inter-subject variability in *fatigue* responses, large amounts of data are needed to better understand the relationship between *fatigue* and non-invasive measures. By enabling remote, continuous monitoring, wearables play an important role in harnessing these data (Park and Jayaraman, 2017, 2021).

## Limitations

To facilitate the identification of studies whose primary aim was to monitor *fatigue*, a limited number of search terms was selected. We are fully aware, that a more open search strategy and the inclusion of MeSH terms could have increased the number of retrieved articles. Nevertheless, we are convinced that the most relevant articles dealing with monitoring of *fatigue* have been detected. Moreover, included studies are of limited comparability, hindering in-depth comparison between proposed *fatigue* monitoring approaches.

Several criteria were defined in the scope of this review due to the lack of well-defined criteria in the existing literature. For instance, datasets were deemed imbalanced when having a samples' proportion more extreme than 60/40. Although arbitrary, those criteria were based on common sense and empirical evidence. Besides, the criterion set to characterized devices as validated or not does not consider aspects regarding devices' reliability and responsiveness. Although they are crucial for device validation, the assessment of device's validity is usually the first step of the validation process. Thus, we are aware that criteria could be further refined.

The present work provides an overview on wearable-based approaches to monitor *fatigue*. While summarizing proposed approaches and major findings, aspects relating to sensors location biases and physiological responses to *fatigue* could not be covered in this review. For instance, although wrist worn GSR sensors for *fatigue* monitoring, there is evidence suggesting that GSR measurements from the wrist are biased indicators of arousal (Tsiamyrtzis et al., 2016). Included studies seldom justify the placement of sensors in the body. In the same way, other factors not covered in this review may have a biasing effect on the reported findings.

## Conclusion

This review has found that the generalizability of much-published research on *fatigue* monitoring through wearables is problematic. The lack of standardization in methods to induce, measure and model *fatigue* limits comparability between studies. A joint effort must be done to find consensus and set adequate standards in this research field.

Research in *fatigue* monitoring through wearables has been focused on the performance of developed measures, while ignoring the underlying mechanisms. Considerably more work will need to be done to design appropriate fatigue-inducing tasks, as well as to study the effect of *fatigue* on parameters derived from physiological/motion signals measured non-invasively.

Ensuring the acquisition of high-fidelity data, by using validated data acquisition systems and implementing signal quality assessment strategies, should be a priority. Ultimately, no accurate and interpretable *fatigue* measure can be developed based on data not representing the concept aimed at. More research is also required to construct measures considering the temporal dynamics of *fatigue*.

Lastly, long-term studies are lacking, which indicates that wearables have not been used to their full potential in *fatigue* research area. Wearables enable continuous, long-term monitoring in an unobtrusive manner. The development of reliable wearable *fatigue* monitoring systems and their implementation in real-world settings creates a unique opportunity to better understand *fatigue* and its impact on subjects' performance.

## Data Availability Statement

The original contributions presented in the study are included in the article/Supplementary Material, further inquiries can be directed to the corresponding author/s.

## Author Contributions

NA, SA, CS, and RR contributed to conception of the manuscript. NA performed the systematic search and data extraction. NA and SA assessed articles for eligibility and made the risk of bias judgments. SA, CS, and RR supervised NA's work. All authors contributed to the development of the risk of bias assessment tool, discussed the results, and contributed to the final manuscript.

## Conflict of Interest

The authors declare that the research was conducted in the absence of any commercial or financial relationships that could be construed as a potential conflict of interest.

## Publisher's Note

All claims expressed in this article are solely those of the authors and do not necessarily represent those of their affiliated organizations, or those of the publisher, the editors and the reviewers. Any product that may be evaluated in this article, or claim that may be made by its manufacturer, is not guaranteed or endorsed by the publisher.
